# Predisposing factors and radiological features in patients with internal carotid artery dissection or vertebral artery dissection

**DOI:** 10.1186/s12883-020-02020-8

**Published:** 2020-12-10

**Authors:** Yongjun Wu, Hongbin Chen, Shihui Xing, Shuangquan Tan, Xinran Chen, Yan Tan, Jinsheng Zeng, Jian Zhang

**Affiliations:** 1 Department of Neurology, The First Affiliated Hospital, Sun Yat-sen University, Guangdong Provincial Key Laboratory of Diagnosis and Treatment of Major Neurological Diseases, National Key Clinical Department and Key Discipline of Neurology, No.58 Zhongshan Road 2, Guangzhou, 510080 China; 2 Department of Neurology, First Affiliated Hospital of Guangxi University of Chinese Medicine, Nanning, Guangxi China

**Keywords:** Craniocervical artery dissection, Carotid artery dissection, Vertebral artery dissection, Cerebral infarction, Ischemic stroke

## Abstract

**Background:**

Cervicocerebral artery dissection is an important cause of ischemic stroke in young and middle-aged individuals. However, very few studies have compared the differential features between internal carotid artery dissection (ICAD) and vertebral artery dissection (VAD), including both cervical and intracranial artery dissections. We conducted a study to investigate the predisposing factors and radiological features in patients with ICAD or VAD.

**Methods:**

All cases diagnosed with cervicocerebral artery dissection, ICAD, or VAD were identified through a medical records database, between January 2010 and January 2020. Baseline characteristics, predisposing factors, and radiological features of ICAD versus VAD were compared.

**Results:**

A total of 140 patients with cervicocerebral artery dissection were included in the study, including 84 patients in the ICAD group and 56 in the VAD group. The mean age of patients in the ICAD and VAD groups was 43.37 ± 14.01 and 41.00 ± 12.98 years old, respectively. Patients with ICAD were more likely to be men compared with VAD (85.71% vs. 67.86%, *p* = 0.012). The frequency of hypertension, diabetes, smoking, drinking, and cervical trauma did not differ between ICAD and VAD. Dissections of ICAD were more frequently at the extracranial portions of the artery compared with those of VAD (70.24% vs. 44.64%, *p* = 0.003). In contrast, dissections of VAD were more common in the intracranial artery (55.36% vs. 29.76%, *p* = 0.003). Radiologically, double lumen (36.90% vs. 19.64%, *p* = 0.029) and intimal flap (11.90% vs. 1.79%, *p* = 0.029) were more frequently observed in ICAD than in VAD, and dissecting aneurysms were less frequent (13.10% vs. 26.79%, *p* = 0.041).

**Conclusions:**

The distributions of cervical and intracranial artery dissections were different between ICAD and VAD. The frequencies of radiological features detected in patients with ICAD and VAD also differed.

## Background

Craniocervical artery dissection (CAD) refers to a tear in the artery lining, resulting in an intramural hematoma toward the intima or the adventitia of the internal carotid artery or vertebral artery [1]. CAD is an important cause of ischemic stroke in young and middle-aged individuals [2, 3]. It has been reported that the annual incidence of spontaneous internal carotid artery dissection (ICAD) is approximately 2.5–3.0 per 100,000 individuals and that of vertebral artery dissection (VAD) is 1.0–1.5 per 100,000 individuals [1, 4]. If no precipitating event can be identified, the dissection is termed “spontaneous”, and causes for the wall lesions are considered to be atherosclerotic changes, inflammation, or genetic disorders (e.g., fibromuscular dysplasia, connective tissue disease). If obvious injuries are identified prior to onset, including minor injuries (e.g., sudden neck rotation and cervical spine manipulation), the dissection is regarded as “traumatic”. At present, the optimal therapy for patients with CAD has not been determined [5]. Antiplatelets or anticoagulants are usually recommended to prevent primary or recurrent ischemic events. However, surgical or endovascular treatments may be used in patients with persistent symptoms of ischemia despite optimum medical treatment, or subarachnoid hemorrhage [6–8].

CAD can affect the cranial or cervical portion of the internal carotid artery or vertebral artery. The predisposing factors, clinical presentation, radiological imaging, and outcome may differ between ICAD and VAD. While most studies investigate these features of ICAD and VAD, relatively few studies actually compare the differential features of ICAD and VAD. In the Cervical Artery Dissection and Ischemic Stroke Patients study, 982 consecutive patients with cervical artery dissection enrolled in European countries were studied and the risk factors, baseline features, and outcomes of ICAD and VAD were compared [4]. As the largest cohort study to update, it has greatly increased our knowledge of the differential features of ICAD and VAD. However, intracranial artery dissection was not included in this study, as intracranial artery dissection occurs less frequently in non-Asian populations [9]. It has been shown that intracranial artery dissection is more likely to affect the posterior circulation, while cervical artery dissection more frequently occurs at the anterior circulation [9–11]. Less information is available about the differential features of ICAD and VAD involving both the cranial and cervical portions of the arteries.

The goal of this study was to compare the baseline characteristics, predisposing factors, clinical findings, and radiological features between ICAD and VAD, including both cervical and intracranial artery dissections.

## Methods

### Participants

**Fig. 1:**
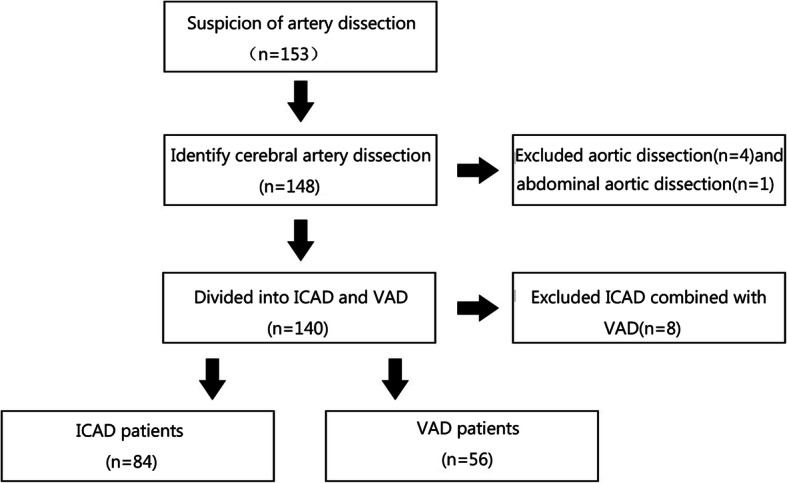
Study flow chart

This retrospective study was approved by the Institutional Review Board of The First Affiliated Hospital of Sun Yat-sen University. Written informed consent regarding the use of data for research purposes was obtained from each patient. All patients diagnosed with artery dissection, CAD, ICAD, or VAD were selected via the medical record system of The First Affiliated Hospital of Sun Yat-sen University, between January 2010 and January 2020 (Fig. 1). The hospital is a national regional medical centre with 3888 beds for the population in South China. A total of 148 cases with a discharge diagnosis of CAD, ICAD, or VAD were included for further evaluation. The diagnosis of CAD, ICAD, or VAD was confirmed by two stroke neurologists and a neuroradiologist based on clinical manifestations and radiological features of the cases. Both cervical artery dissection and intracranial artery dissection were included in the analysis if patients presented with an intramural hematoma, intimal flap, double lumen, long tapering stenosis, artery occlusion that recanalizes in an irregular aneurysmal dilation or stenosis, or irregular aneurysmal dilation with rapid changes in morphology or associated with focal stenosis on magnetic resonance imaging and angiography (MRI, MRA), or computed tomography angiography (CTA), or digital subtraction angiography (DSA) [9, 12, 13]. Patients were excluded if they had co-occurrence of CAD and VAD, or if they had dissection for iatrogenic causes, or if dissection or injury of other supra-aortic arteries were identified, including aortic dissection.

### Predisposing factors

Demographic data and potential predisposing factors were extracted from the medical records, including age, sex, hypertension, diabetes, alcohol drinking, smoking, cervical massage, and head or cervical trauma. Total cholesterol, triglycerides, low-density lipoprotein cholesterol (LDL-C), high-density lipoprotein cholesterol (HDL-C), and blood glucose levels were also collected. Hypertension was defined as a history of systolic blood pressure ≥ 140 mmHg and/or diastolic blood pressure ≥ 90 mmHg, or if the patient was taking antihypertensive drugs. Diabetes was defined by a history of fasting blood glucose ≥7.0 mmol/L, 2 h postprandial ≥11.1 mmol/L, or the use of hypoglycemic therapy. All data were measured within 48 h of hospitalisation. We defined current smokers as individuals who smoked any tobacco in the past 12 months and included those who had quit within the past year. Alcohol consumption was recorded as: past or present drinking (more than 1 drink per month) [14]. A history of preceding trauma was defined as a physical effect on the head or neck (e.g., abrupt cervical movements, cervical manipulation, sporting activities, impact injury of the head and neck) within 1 month before the onset of artery dissection [15].

### Radiological features

All patients underwent a standard brain MRI on a 3.0 T MRI system (MagnetomVerio, Siemens, Erlangen, Germany). Cervical and cranial vascular imaging was performed with MRA, CTA, or DSA. Radiological presentations of dissections and the sites of infarct or hemorrhage were identified by two stroke experts and a neuroradiologist. If any disagreement arose, a discussion was conducted until a consensus was reached. Confirmed CAD was diagnosed based on the following criteria: 1. The presence of intramural hematoma, intimal flap (a piece of intima torn by dissection project into the lumen of the artery), double lumen; 2. Long tapering stenosis (stenosis characterized by a long segment of narrowing of artery) with a fusiform or irregular aneurysmal dilation, or a rapid change in morphology on repeated imaging; 3. Occlusion recanalizes in either an irregular aneurysmal dilation or a long filiform or irregular stenosis [9, 12, 13].

### Treatment

The treatment of patients with CAD was recorded, including medical treatment and surgical or endovascular procedures. The severity of stroke and any functional impairment at admission was evaluated using the National Institutes of Health Stroke Scale (NIHSS) and modified Rankin Scale Score (MRS), respectively. The NIHSS and MRS were retested 2 weeks after the onset of symptoms.

### Statistical analysis

The predisposing factors, clinical features, and radiological features were compared between the ICAD and VAD groups. Quantitative data of normal distribution were expressed as mean ± standard deviation (Mean ± SD) and analyzed using the student’s t test. Quantitative data of skewed distribution were presented as median and quartile; and analyzed using the Wilcoxon rank-sum test. Qualitative data were expressed as frequency and percentage (%), and the chi-square test or Fisher’s exact test was used for statistical difference analysis. All data were analyzed using GraphPad Prism Version 8.0 software (Graph Pad Software Inc., San Diego, CA, USA). A *p*-value of< 0.05 was considered statistically significant.

## Results

### Predisposing factors distribution

A total of 148 patients with CAD were identified in this study. Eight cases were excluded because of the concurrence of ICAD and VAD, including two cases of fibromuscular dysplasia. In the remaining 140 patients, 110 were male (78.7%) and 30 were female (22.3%). The mean age was 42.1 years (range = 5–80 years). Patients were divided into two groups, including 84 patients in the ICAD group and 56 patients in the VAD group. The mean age of patients with ICAD and VAD was 43.37 ± 14.01 and 41.00 ± 12.98 years old, respectively. No significant difference in age was found between the two groups (Table 1). There were 72 (85.71%) men in the ICAD group and 38 (67.86%) in the VAD group, indicating that patients with ICAD were more likely to be men (*p* = 0.012, Table 1). The frequencies of hypertension, diabetes, smoking, and drinking were similar in patients with ICAD and VAD. Nine (10.71%) patients with ICAD and 5 (8.93%) with VAD had a history of trauma to the head or neck. In addition, five (5.95%) of the ICAD cases and 3 (5.36%) of the VAD cases underwent cervical spine manipulation in the preceding month. No significant difference in cervical trauma was observed between the two groups (Table 1). One patient with fibromuscular dysplasia was identified in VAD, but the incidence of fibromuscular dysplasia did not differ between ICAD and VAD (*p* = 0.219, Table 1). There was also no significant difference in blood glucose and lipid levels, including triglycerides, LDL-C, and HDL-C.

**Table 1:** Predisposing factors

Predisposing factors	ICAD(*n* = 84)	VAD(*n* = 56)	OR(95%CI)	*P* value
Age, years mean ± SD	43.37 ± 14.01	41.00 ± 12.98	0.99 (0.96-1.01)	0.315
Male sex (%)	72 (85.71%)	38 (67.86%)	2.84 (1.27-6.28)	0.012
Hypertension (%)	22 (26.19%)	12 (21.43%)	1.30 (0.58-3.01)	0.520
Diabetes (%)	3 (3.57%)	3 (5.36%)	0.65 (0.15-2.89)	0.609
Smoking (%)	32 (38.10%)	20 (35.71%)	1.11 (0.55-2.31)	0.775
Drinking (%)	27 (32.14%)	13 (23.21%)	1.57 (0.75-3.36)	0.252
Head or neck trauma (%)	9 (10.71%)	5 (8.93%)	1.22 (0.39-3.41)	0.730
Cervical manipulation (%)	5 (5.95%)	3 (5.36%)	1.12 (0.28-4.36)	0.882
Fibromuscular dysplasia (%)	0 (0%)	1 (1.79%)	0.22 (0.01-5.48)	0.219
History of migraine(%)	2 (2.33%)	4 (7.14%)	0.31 (0.06-1.38)	0.163
Total cholesterol (mmol/L)	4.12 ± 1.26	4.24 ± 1.17	1.04 (0.83-1.31)	0.584
Triglycerides (mmol/L)	1.46 ± 0.88	1.33 ± 0.70	0.83 (0.55-1.27)	0.396
HDL-C (mmol/L)	1.05 ± 0.22	1.10 ± 0.26	1.55 (0.54-4.47)	0.191
LDL-C (mmol/L)	2.56 ± 0.99	2.66 ± 0.89	1.07 (0.78-1.47)	0.552
Blood glucose (mmol/L)	5.11 ± 1.59	5.38 ± 1.73	1.03 (0.85-1.25)	0.340

*HDL-C* high-density lipoprotein cholesterol, *LDL-C* low-density lipoprotein cholesterol

### Radiological features analysis

Of the 140 patients with CAD, 56 patients (40%) had an intracranial dissection and the remaining 84 patients (60%) had an extracranial dissection. The dissections of 18 cases (12.86%) extended from the extracranial to intracranial artery (Table 2). Of the ICAD cases, 47 cases (55.95%) were left-sided, 32 (38.1%) were right-sided, and 5 (5.95%) were bilateral. There were more left-sided dissections in patients with ICAD than in those with VAD (55.95% vs. 30.36%, *p* = 0.003, Table 2). In addition, the dissections were more frequently identified in the extracranial portions of the artery compared with those of VAD (70.24% vs. 44.64%, *p* = 0.003, Table 2). In contrast, dissections of VAD were more common in the intracranial artery (55.36% vs. 29.76%, *p* = 0.003).

**Table 2:** Radiological features

Imaging	Total(*n* = 140)	ICAD(*n* = 84)	VAD(*n* = 56)	OR(95%CI)	*P* value
Intracranial dissection	56 (40.00%)	25 (29.76%)	31 (55.36%)	0.34 (0.18-0.68)	0.003
Extracranial dissection	84 (60.00%)	59 (70.24%)	25 (44.64%)	2.93 (1.46-5.69)	0.003
Intracranial extension	18 (12.86%)	11 (13.10%)	7 (12.50%)	1.06 (0.37-2.73)	0.918
Left-sided	64 (45.71%)	47 (55.95%)	17 (30.36%)	2.91 (1.45-5.92)	0.003
Right-sided	60 (42.86%)	32 (38.10%)	28 (50.00%)	0.62 (0.31-1.20)	0.163
Bilateral	9 (6.43%)	5 (5.95%)	4 (7.14%)	0.82 (0.23-2.79)	0.778
Vessel occlusion	26 (18.57%)	19 (22.62%)	7 (12.50%)	2.05 (0.81-5.17)	0.132
Dissecting aneurysms	26 (18.57%)	11 (13.10%)	15 (26.79%)	0.41 (0.18-0.98)	0.041
Double lumen	42 (30.00%)	31 (36.90%)	11 (19.64%)	2.39 (1.12-5.08)	0.029
String sign	29 (20.72%)	16 (19.05%)	13 (23.21%)	0.78 (0.35-1.81)	0.551
Intimal flap	11 (7.85%)	10 (11.90%)	1 (1.79%)	7.43 (1.19-82.16)	0.029
Intramural hematoma	32 (7.14%)	20 (23.81%)	12 (21.43%)	1.45 (0.50-2.69)	0.742
Total cerebral infarct	118 (84.29%)	72 (85.71%)	46 (82.14%)	1.30 (0.50-3.16)	0.570
TIA	5 (3.57%)	4 (4.16%)	1 (1.79%)	2.75 (0.43-34.19)	0.353
SAH	4 (2.86%)	2 (2.38%)	2 (3.57%)	0.66 (0.11-4.32)	0.679
Basal ganglia	38 (27.14%)	38 (45.24%)	0 (0%)	--(11.611- --)	<0.001
Frontal lobe	38 (27.15%)	38 (45.24%)	0 (0%)	--(12.80- --)	<0.001
Parietal lobe	30 (21.43%)	30 (35.71%)	0 (0%)	--(8.53- --)	<0.001
Temporal lobe	26 (18.57%)	26 (30.95%)	0 (0%)	--(6.81- --)	<0.001
Occipital lobe	10 (7.14%)	10 (11.90%)	0 (0%)	--(2.12- --)	<0.001
Thalamus	2 (1.43%)	0 (0%)	2 (3.57%)	0.66 (0.10-4.32)	0.081
Cerebellum	13 (9.29%)	0 (0%)	13 (23.21%)	0.00 (0.00-0.16)	<0.001
Medulla oblongata	21 (15.00%)	0 (0%)	15 (37.5%)	0.00 (0.00-0.08)	<0.001
Pons	8 (5.71%)	0 (0%)	8 (14.29%)	0.00 (0.00-0.28)	<0.001
Hemorrhagic transformation	18 (15.25%)	11 (15.28%)	7 (15.22%)	1.06 (0.37-2.73)	0.993

*TIA* transient ischemic attack, *SAH* subarachnoid hemorrhage

A typical feature of dissection was identified by radiological studies. The most common imaging feature of dissection was the double lumen (30%), followed by string sign, occlusion, and dissecting aneurysms. Double lumen (36.90% vs. 19.64%, *p* = 0.029, Table 2) and intimal flap (11.90% vs. 1.79%, *p* = 0.029) were more frequent in patients with ICAD, while dissecting aneurysms less frequently (13.10% vs. 26.79%, *p* = 0.041, Table 2). However, the vessel occlusion, string sign, and intramural hematoma did not differ between the two groups (Table 2).

Most of the CAD cases presented as ischemic events, including cerebral infarction (84.29%) and transient ischemic attack (3.57%). Subarachnoid hemorrhage accounted for 2.86% of all CAD cases. No difference was found in ischemic events or subarachnoid hemorrhage between ICAD and VAD (Table 2). The infarct was identified on MRI in 85.71% of ICAD and 82.14% of VAD. Of the ICAD cases, 38 (45.24%) were in the basal ganglia, 38 (45.24%) were in the frontal lobe, 30 (35.71%) were in the parietal lobe, and 26 (30.95%) were in the temporal lobe. Most of the infarctions in VAD cases were in the medulla oblongata (37.5%), cerebellum (23.71%), and pons (14.29%). A total of 18 patients with CAD and ischemic stroke underwent hemorrhagic transformation. The proportion of hemorrhagic transformation did not differ between ICAD and VAD (Table 2).

### Treatment

Patients were treated in the neurological stroke unit or neurosurgery department. Intravenous thrombolysis was administered 4.5 h after onset according to the guidelines for management of acute ischemic stroke. Other treatments included endovascular therapy, anticoagulation, and antiplatelet therapy (aspirin, clopidogrel, and aspirin/clopidogrel). Six patients received intravenous thrombolysis with alteplase. Another 14 patients were treated with endovascular stent implantation due to severe stenosis, intraluminal thrombosis, or aneurysm, including 10 ICAD and 4 VAD cases. Four patients received anticoagulant therapy with rivaroxaban, and 136 patients were administered aspirin, clopidogrel, or dual antiplatelets as secondary prevention strategies. The severity of the stroke and functional impairments were evaluated using NIHSS and MRS, at both admission and discharge. Patients with ICAD suffered from a more severe stroke and greater impairment of neurological function at admission (*p* = 0.041, *p* = 0.011, Table 3). However, the NIHSS and MRS results between the two groups after 2 weeks did not differ significantly. Most of the patients with CAD had a favorable outcome at discharge (Table 3).

**Table 3:** Treatments and stroke severity

	ICAD(*n* = 84)	VAD(*n* = 56)	OR(95%CI)	*P* value
Intravenous thrombolysis	4 (4.76%)	2 (3.57%)	1.35 (0.31-7.28)	0.733
Anticoagulation	4 (4.76%)	0 (0%)	6.32 (0.33-119.8)	0.098
Anti-platelet	80 (95.24%)	56 (100%)	0.16 (0.01-3.00)	0.098
Endovascular procedures	10 (11.90%)	4 (7.14%)	1.96 (0.64-5.83)	0.358
Initial NIHSS	6.54 ± 5.93	4.66 ± 5.67	0.94 (0.89-1.00)	0.041
NIHSS at discharge	3.85 ± 4.06	3.45 ± 4.82	0.98 (0.90-1.06)	0.598
Initial MRS (≤2)	55 (65.48%)	47 (83.93%)	0.36 (0.15-0.83)	0.016
MRS(≤2) at discharge	69 (82.14%)	50 (89.29%)	0.55 (0.20-1.44)	0.246

*NIHSS* National Institutes of Health Stroke scale, *MRS* modified Rankin Scale

## Discussion

It is well recognized that CAD is a major cause of ischemic stroke in young adults. In this study, the mean age of 140 patients with CAD was 42 years, which was similar to previous reports [2, 3]. The CADSIP study and an earlier study demonstrated that most CAD cases were likely to be male, especially in patients with ICAD [4, 16]. Our data showed that 110 (78.7%) patients with CAD were male and that patients with ICAD were more often men. The reasons for the higher prevalence of ICAD in male remain unknown. It is speculated that genetic factors, hormones, and the difference in strength of neck muscles and dynamic stabilization during head acceleration may play a role [16]. In contrast to previous studies that suggested patients with ICAD were older and more frequently experienced cervical trauma [4, 17], we did not find any differences in age, head or neck trauma, or cervical manipulation between patients with ICAD versus VAD. This discrepancy may result from the inclusion of more patients with intracranial artery dissection in our study.

Although a history of cervicocerebral trauma was more often present in patients with cervical artery dissection [18, 19], it has not yet been established as a risk factor for intracranial artery dissection [9]. Moreover, the distribution of vascular risk factors such as hypertension, diabetes, hypercholesterolemia, smoking, and drinking did not differ between the two groups. These results were consistent with the CADSIP study. These observations suggest that these vascular risk factors may affect both carotid artery and vertebral artery. Genetic factors may also play an important role in the pathogenesis of CAD. It has been reported that CAD may be a complication of monogenic connective tissue diseases, such as vascular Ehlers-Danlos syndrome [20] and Marfan syndrome [21]. In addition, some studies have shown that variations in the ICAM-1 [22] (intercellular adhesion molecule 1) and MTHFR [23] (methylenetetrahydrofolate reductase) genes may be associated with CAD. Unfortunately, genetic screening was not performed in our patients. The influence of genetic factors on the intracranial and cervical artery dissections is unknown. Fibromuscular dysplasia, a non-atherosclerotic and non-inflammatory vascular disease, has also been demonstrated to increase the recurrence of CAD [24]. Three cases with fibromuscular dysplasia were identified in our series, but two were excluded due to the concurrence of ICAD and VAD. Consistent with previous studies, the frequency of fibromuscular dysplasia did not differ between ICAD and VAD cases [25]. However, more studies are needed to further explore the genetic factors associated with CAD.

In this study, intracranial artery dissection accounted for 40% of all CAD cases. More interestingly, the dissections of ICAD were often located at the extracranial portion of the internal carotid artery (70.24%), while those of VAD were more often at the intracranial segments of the vertebral artery (55.36%). In parallel to the involvement of the intracranial portion in the majority of VAD cases, most of the infarctions in VAD were present in the medulla oblongata and cerebellum. The high proportion of intracranial artery dissection in our study may represent the influence of ethnic origin and our enrollment strategy. In a European study that prospectively recruited patients in the neurology department, intracranial VAD was reported to account for only 11% of all VAD cases [26]. In contrast, intracranial VAD was found to be as high as 90% in a Japanese study based on data collected from the hospital database system [27]. In another retrospective study of a Korean population, intracranial CAD accounted for about 78% of all cases of CAD [28]. Taken together, these studies suggest that the East Asian population may have a higher incidence of intracranial artery dissection than the European population. Retrospective studies involving both the neurology and neurosurgery departments may also result in a higher proportion of intracranial CAD. Future population-based studies are needed to elucidate whether ethnic origin or recruitment bias affects the location of dissection.

DSA is regarded as the gold standard for the depiction of CAD, as it can directly display typical features of dissection, such as intimal flaps, string signs, vessel stenosis, and occlusion [13, 29]. However, DSA is an invasive modality with potential complications. It is not suitable for application in all patients. Instead, CTA and MRA are considered non-invasive imaging approaches to detect CAD and can be used in most patients [12, 13, 30]. Unfortunately, these modalities of angiography are not able to detect intramural hematoma. In this instance, T1-weighted axial MRI scan with fat-suppression technique has a high sensitivity and specificity, and is recommended for the diagnosis of CAD, in combination with MRA, to visualize not only the arterial lumen but also arterial wall deficits [6]. Recently, high-resolution MRI and three-dimensional acquisition of fat-suppressed sequences with black-blood effects have further improved the detection of CAD. This sequence uses the double inversion-recovery technique to null blood signals, thereby providing a directed lineation of the arterial wall [31–33]. It can clearly visualize the intimal flap, true and false lumen, as well as intramural hematoma [32, 33]. In this study, CAD was diagnosed using T1-weighted and T2-weighted MRI, combined with MRA, CTA, or DSA. We found that double lumen (36.90%) and intimal flap (11.90%) were more common in ICAD, while dissecting aneurysms occurred more frequently in VAD (26.79%). One possible explanation for the greater visualization of the double lumen and intimal flap in ICAD is the larger diameter of the internal carotid artery. The small diameter and great variability of the vertebral artery may limit the detection of dissection using standard MRI and angiography. With the use of high-resolution MRI, the detection of double lumen and intimal flap in the vertebral artery could be increased [33]. More observation of dissecting aneurysms in VAD could result from a higher prevalence of detected intracranial artery dissection. Compared with the cervical artery, the intracranial artery is short of elastic fibres in the media and adventitial tissue, and has no external elastic layer [4, 34]. Therefore, intracranial dissection is more likely to develop dissecting aneurysms due to the deficiency of supporting tissue in the vessel. On the other hand, the different frequency of double lumen, intimal flap, and dissecting aneurysms may suggest that dissections of the internal carotid artery are more often subintimal, while those of the vertebral artery are more prone to being subadventitial [9]. The higher prevalence of dissecting aneurysms in VAD should be taken into account when choosing therapeutic strategies. It has been reported that patients with intracranial artery dissection have a higher risk of developing subarachnoid hemorrhage [35]. In this circumstance, the use of antiplatelets and anticoagulants should be cautioned.

Consistent with previous studies [4, 36], on admission, patients with ICAD experienced a more severe stroke than those with VAD. This result was also in line with more frequent complaints of hemiparesis and aphasia in ICAD, which formed part of the NIHSS assessment. In contrast, some common symptoms of VAD, such as dizziness, nausea, and vomiting, were not evaluated in the NIHSS. Therefore, the severity of VAD may be underestimated. Overall, the majority of patients with ICAD or VAD experienced a mild to moderate stroke, as indicated by relatively low NIHSS scores. Most of the studies, including our results, suggested that the majority of patients with CAD have a favorable outcome [4, 11, 17, 36].

The main limitation of this study is the retrospective design based on a single center and lack of control. Asymptomatic or mild cases could have been underestimated in the present study, and referral bias should also be considered. Second, although our results suggest that patients with CAD have a favorable outcome at discharge, follow-up data were not available, and any recurrence of dissections and strokes were not known.

## Conclusions

The distribution of cervical and intracranial artery dissections were different between ICAD and VAD. The frequencies of radiological features such as double lumen, intimal flap, and dissecting aneurysms also differed in patients with ICAD and VAD.

## Data Availability

The data sets in this study are available from the corresponding author on reasonable request.

## Abbreviations

CAD: Cervicocerebral artery dissection
ICAD: Internal carotid artery dissection
VAD: Vertebral artery dissection
CADISP: Cervical artery dissection and ischemic stroke patients
NIHSS: National institutes of health stroke scale
MRI: Magnetic resonance imaging
MRA: Magnetic resonance angiography
CTA: Computed tomography angiography
DSA: Digital subtraction angiography
LDL-C: Low-density lipoprotein cholesterol
HDL-C: High-density lipoprotein cholesterol
MRS: Modified rankin scale score.
